# The role of topical therapies in the treatment of Chronic Rhinosinusitis

**DOI:** 10.1590/S1808-86942011000600001

**Published:** 2015-10-19

**Authors:** Jeffrey D. Suh, Vijay Ramakrishnan, Alexander G. Chiu

**Affiliations:** Division of Otolaryngology - Head and Neck Surgery, UCLA School of Medicine, Los Angeles, CA 90095; Department of Otolaryngology, University of Colorado School of Medicine, Aurora, Colorado 80045; Division of Otolaryngology - Head and Neck Surgery, University of Arizona, Tucson, AZ 85724

Chronic rhinosinusitis (CRS) is a common disease resulting from inflammation of the sinonasal mucosa. The underlying cause of the inflammation is multifactorial, with both genetic and environmental contributions^1^. Guidelines published by the AAOHNS in 2007 and a Cochrane review by Harvey *et al.* published in 2009 have clearly established the benefits of nasal saline irrigation for patients with CRS^1^,^2^. Benefits include improved mucociliary function, decreased nasal mucosal edema, and mechanical removal of infectious debris, allergens, mucus, and bacteria from the sinus and nasal cavities. Irrigation may also reduce reliance on other sinus medications, the number and frequency of acute exacerbations of CRS, and symptoms related to CRS. Side effects of saline irrigation are minimal and self-limiting, mostly related to local irritation or ear symptoms.

Nasal Irrigation has also been recognized as a potential route for topical drug administration into paranasal sinuses due to ease of use and direct drug effects on the sinonasal mucosa. Topical drugs being investigated for use in CRS include antimicrobial, anti-inflammatory and immunomodulatory agents. One difficult question for rhinologists to answer is w*hich patients with rhinosinusitis are the best candidates for topical approaches?* Desired properties of all topical therapies include complete sinus distribution, high local drug absorption, low systemic absorption, and minimal toxicity to the cilia and sinonasal mucosa. Understandably, the benefits of saline irrigation and topical therapies are greatest in patients who have had prior functional endoscopic sinus surgery (FESS)^3^,^4^. Clinical studies support this finding, with distribution of topical solutions in the unoperated sinuses on the order of less than 2% of the total irrigation volume, with almost no penetration in the frontal and sphenoid sinuses^4^. For those patients with mucosal edema from infection and chronic inflammation, distribution is probably significantly less.

Oral and intravenous antimicrobial therapies have traditionally been prescribed to manage infectious exacerbations of CRS. However, these agents are not without significant side effects, especially for patients requiring prolonged courses. Topically applied antibiotics in irrigation have a theoretical advantage to localize high doses of antibiotics to the sinonasal mucosa while minimizing systemic side effects. The majority of clinical data for topical antibiotics has investigated and provided support for the use of mupirocin sinus irrigation for patients with MRSA infections^5^,^6^. Our lab investigated the use of topical tobramycin in a rabbit model of *Pseudomonas* sinusitis, and found that topical tobramycin resulted in the eradication of viable bacteria within the lumen of the sinus. However, even at high concentrations, topical tobramycin could not eradicate *Pseudomonas* attached to the mucosa in biofilms^7^. Potential side effects of topical antibiotic therapy, such as vertigo or hearing loss, can be caused by either direct exposure to the middle ear or via systemic toxicity. Research has demonstrated systemic absorption and potential ototoxicity with aminoglycoside irrigation at commonly used concentrations^8^. For the antibacterial washes, the highest level of evidence currently exists for use in postsurgical patients and culture-directed therapy. However, despite the popularity and emergence of topical antibiotics for CRS as a treatment modality, there is currently only low-level evidence (Level III) supporting its use.

Biofilms have been identified as a potential risk factor for worse outcomes after endoscopic sinus surgery, persistent sinonasal inflammation, and a cause of recalcitrant infections^9^,^10^. Traditional medical management for CRS is largely inadequate to eradicate sinonasal biofilms. Several investigations have recently reported on topical agents to combat mucosal biofilms. Topical agents specifically proposed for the treatment of sinonasal biofilms in CRS have included mupirocin^6^, tobramycin^11^, moxifloxacin^12^, antimicrobial peptides^13^, iron chelators^14^, Manuka Honey^15^, and surfactants^12^,^14^,^16^,^17^. *In vitro* studies by Desrosiers *et al*. and Ha *et al*. were able to eliminate *S. aureus* biofilms with antibiotics in concentrations easily obtainable in topical solutions^12^,^18^. However, enthusiasm for many of these therapies has been tempered by studies demonstrating by biofilm regrowth after treatment and ciliotoxic effects.

Surfactants in nasal irrigation have been shown to be effective for some patients with CRS. Surfactants are thought to improve mucocilliary clearance by reducing the adherence of sputum to the epithelial layer which increases efficiency of energy transfer from the cilia to the mucus layer. We previously reported on the use of commercially available dilute baby shampoo irrigation in 18 patients with recalcitrant CRS. Almost half of patients in this study experiencing an overall improvement in their subjective symptoms, and 60% noting reductions in postnasal drainage and thick mucus^17^. More recent investigations from our lab of a similar topical surfactant solution showed no ciliotoxicity or epithelial injury^19^. Future prospective, blinded and controlled studies are required to better understand the role of baby shampoo or other surfactants in the treatment of CRS.

Topical and oral steroids are commonly used to reduce sinonasal mucosal inflammation and to treat nasal polyps. Use of steroid irrigation has become more common to spare patients from the side effects and toxicities of systemic steroids, while providing higher concentrations of steroid directly to the sinus mucosa than what is possible with topical nasal sprays. Various off-label mixtures have been described for irrigation, including mixtures of budesonide with saline at various concentrations^20^. Studies have shown that short-term use of steroid washes are well tolerated, without suppression of the hypothalamicpituitary-adrenal (HPA) axis^21^,^22^.

Despite increasing use of topical therapies for CRS, many questions remain regarding the optimal length of treatment, optimal delivery mechanism, and long-term effects of the treatments on the sinonasal mucosa and cilia. In addition, treatments with topical antibiotics, corticosteroids, and surfactants in combination could show synergistic benefits depending on the specific clinical presentation. Future studies will undoubtedly shed light on these questions, and provide more evidence of the efficacy of topical therapies for CRS.
